# Simultaneous application of BrdU and WST-1 measurements for detection of the proliferation and viability of airway smooth muscle cells

**DOI:** 10.1186/0717-6287-47-75

**Published:** 2014-12-22

**Authors:** Lei-Miao Yin, Ying Wei, Wen-Qian Wang, Yu Wang, Yu-Dong Xu, Yong-Qing Yang

**Affiliations:** Laboratory of Molecular Biology, Shanghai Research Institute of Acupuncture and Meridian, Yue Yang Hospital, Shanghai University of Traditional Chinese Medicine, Shanghai, 200030 China

**Keywords:** BrdU, WST-1, Cell proliferation, Cell viability, Airway smooth muscle cells

## Abstract

**Background:**

BrdU is a commonly used reagent in cell proliferation assays, and WST-1 measurement is widely used to detect cell viability. However, no previous study has formally reported the combination of the two assays, which may be used to detect the proliferation and viability simultaneously. In this study, we examined the effect of adding BrdU 2 h prior to the WST-1 assay and tried to test the possibility of the combined detection using rat airway smooth muscle cells.

**Results:**

The WST-1 measurements obtained from the combined detection were consistent with those obtained from the separate detection, which suggested that the addition of BrdU 2 h prior to the WST-1 analysis did not affect the WST-1 results. The BrdU measurements obtained from the combined detection also demonstrated the same trend as that obtained from the separate detection, and dosages of 200, 400 and 800 ng/ml testing reagent significantly inhibited the proliferation of rat airway smooth muscle cells.

**Conclusions:**

Our study suggests that the BrdU and WST-1 measurements can be applied simultaneously without mutual interference, which may increase the efficacy and consistency of these measurements to a certain extent.

## Background

Proliferation is an important characteristic of living cells, and increases in cell numbers are the result of cell division. Many methods have been established for cell number quantification, and all of these have underlying assumptions, relative merits, and must be applied appropriately. Both direct and indirect techniques have been used for measuring cell proliferation. The counting of cell numbers using a hemocytometer and the clonogenic assay are the classical methods used for direct measurement [1]. The indirect measurement methods included isotope-related methods and non-radioactive assays. Isotopes, such as ^3^H, ^76^Br, ^15^ N and ^13^C, are used to detect cell proliferation, but radioactive products and/or special facilities are required. Non-radioactive assays are widely used because of lower toxicity, convenient operation and low cost. Alamar blue has been adopted to determine the proliferation of different cell types [2]. A highly sensitive, fluorescence-based microplate assay has also been applied for cell number determination [3]. The in situ hybridization of histone mRNA and immunohistochemistry with Ki-67 are preferable techniques for assessing cell proliferation in paraffin-embedded renewing tissues [4]. The expression levels of BrdU (Bromodeoxyuridine), Ki-67, and PCNA (proliferating cell nuclear antigen) have been compared by immunohistochemical labeling to evaluate the proliferation of epidermal basal cells, and the results showed that there was no substantial difference among the three methods [5].

As a commonly used reagent in cell proliferation assay, BrdU is a thymidine analog that can incorporate into the newly replicated DNA of S-phase cells. BrdU has a high labeling efficiency and can be detected within a short time. It is reported that the labeling efficiency of BrdU was 94% in bone marrow stromal cells [6] and approximately 10% of the BrdU-labeled mesenchymal stem cells could still be detected after 25 d [7]. By using BrdU, the newly synthesized DNA in primary murine hippocampal neurons could be labeled within 4 h [8] and the generation time of gastrulating chick embryos was found to range from 2 h to 10 h [9]. Moreover, various methods can be used to apply the BrdU reagent. The proliferation of hippocampal progenitor cells has been quantified by labeling with 50 mg/kg BrdU through intraperitoneal injection [10]. The injection of 200 mg BrdU through the intravenous route has been used to evaluate the biological role of the HER (human epidermal growth factor receptor) gene family of receptor tyrosine kinases [11].

The BrdU labeling method is compatible with other methods. It is reported that the RNA quality of the stained section could be preserved after BrdU immunostaining in bovine mammary tissue [12]. However, the combination of BrdU labeling with WST-1 (2-(4-iodophenyl)-3-(4-nitrophenyl)-5-(2,4-disulfophenyl)-2H-tetrazolium, mono-sodium salt), which is frequently used to detect cell viability, has not been formally investigated. The examination of the cell proliferation and viability of the same cell population will double the data and reduce the amount of work, which may also result in saving expensive testing reagents and labor. In this study, we examined the effect of adding BrdU 2 h prior to the WST-1 assay and tried to test the possibility of the combined detection.

## Results

**Figure 1:**
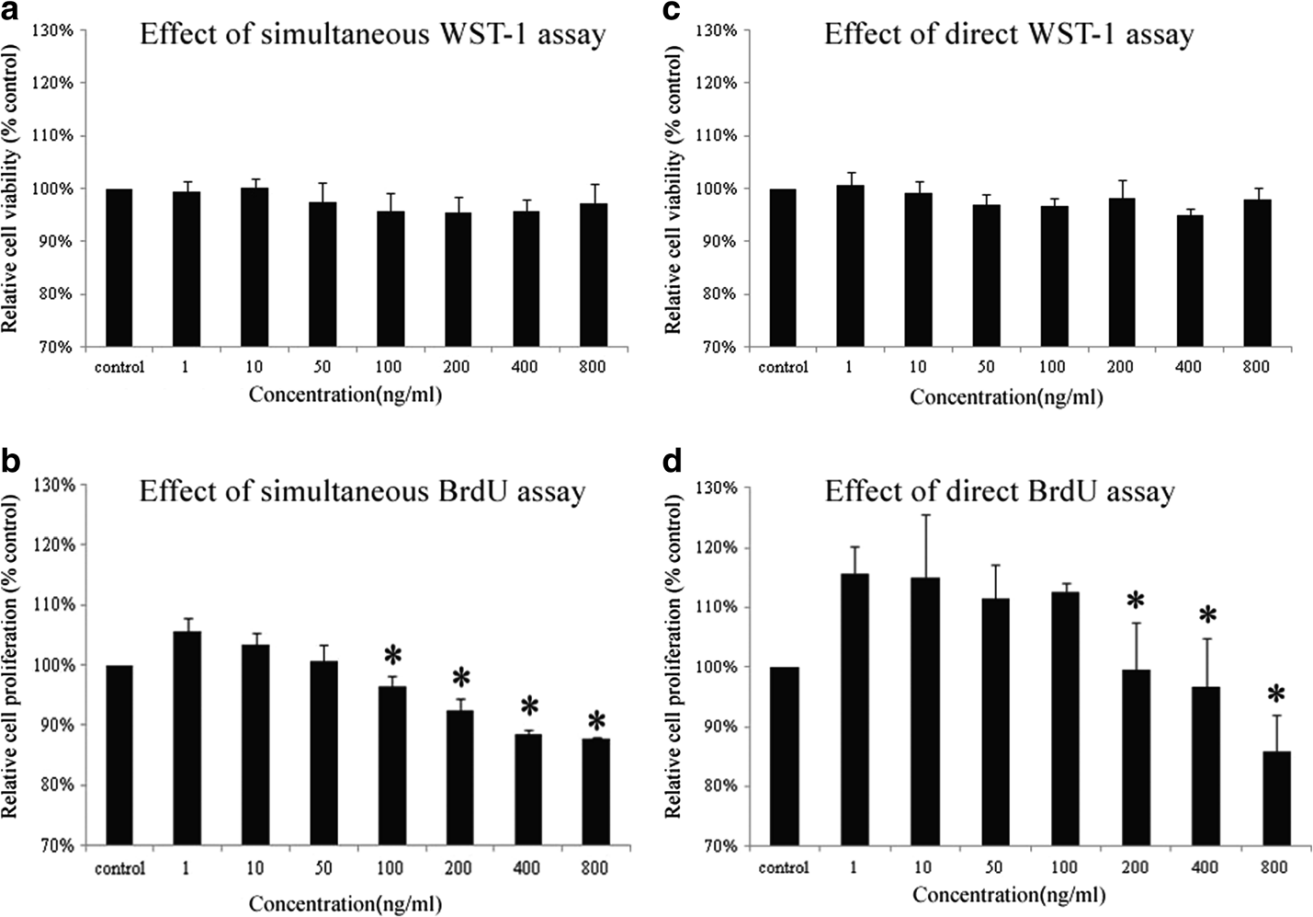
**The comparison between the simultaneous and direct assays. a)** The WST-1 measurement obtained 2 h after the addition of BrdU showed that there was no significant difference among the different dosages of the MRP-14 protein (P > 0.05). **b)** The BrdU measurement performed after WST-1 assay showed that there was a significant difference among the different groups (P < 0.05). The protein dosages of 100, 200, 400 and 800 ng/ml significantly inhibited the proliferation of ASM cells at 24 h (P < 0.05). **c)** The WST-1 measurement was conducted directly 24 h after the addition of the MRP-14 protein at concentrations of 1, 10, 50, 100, 200, 400 and 800 ng/ml. The result manifested that there was no significant difference among the different dosages of the MRP-14 protein (P > 0.05). **d)** The direct BrdU measurement was obtained 24 h after the addition of the MRP-14 protein at concentrations of 1, 10, 50, 100, 200, 400 and 800 ng/ml. The result showed that the protein concentrations of 200, 400 and 800 ng/ml significantly inhibited the proliferation of ASM cells at 24 h (P < 0.05). *indicates a significant difference compared with the result obtained with 1 ng/ml (P < 0.05). All of the data were expressed as mean ± SD, n = 4.

After adding the BrdU into the 96-well plate for 2 h, the WST-1 measurement was performed. The WST-1 result (combined) showed that there was no significant difference among the different dosages of the MRP-14 protein (F = 1.617, P > 0.05, Figure 1a), although certain dosages showed the tendency of inhibition on the ASM cells. After the WST-1 measurement, the BrdU assay was then conducted. The result showed that there was a significant difference among the different groups (F = 60.586, P < 0.05). The dosages of 100, 200, 400 and 800 ng/ml protein inhibited the proliferation of the ASM cells at 24 h and were significantly different from that of the 1 ng/ml (P < 0.05, Figure 1b). The results also demonstrated that low concentrations of the MRP-14 proteins (1, 10 ng/ml) had the tendency to stimulate cell growth.

At the same time, the standard WST-1 measurement was conducted directly after the addition of the same concentrations of the MRP-14 protein. The WST-1 result (standard) demonstrated that there was no significant difference among the different dosages of the MRP-14 protein (F = 2.443, P > 0.05, Figure 1c). The fact suggested that the addition of BrdU 2 h prior to the WST-1 examination did not disturb the WST-1 result and thus provided a possibility for the combined detection. In order to confirm that the proliferation status of the ASM cells was not disturbed by the WST-1 reagent, the BrdU measurement was performed directly 24 h after the addition of the same concentrations of the MRP-14 protein. The result showed that there was a significant difference among the different groups (F = 10.559, P < 0.05). The results obtained with dosages of 200, 400 and 800 ng/ml were significantly different from that obtained with 1 ng/ml (P < 0.05, Figure 1d), which suggested that these dosages of the MRP-14 protein inhibited the proliferation of ASM cells at 24 h. The 1 and 10 ng/ml MRP-14 protein stimulated the proliferation of ASM cells, which were consistent to the previous experiment.

## Discussion

BrdU was first introduced to examine cell proliferation in the central nervous system in 1988 and is useful for labeling nascent DNA in living cells and tissues [13, 14]. WST-1 is a cell-impermeable, sulfonated tetrazolium salt that can be used to examine cell viability without killing the cells [15]. It will be cost-efficient to combine the BrdU and WST-1 measurements for the simultaneous detection of cell proliferation and viability.

The low concentration and short application time of BrdU are the first components that allows its possible combination with the WST-1 assay. Early study showed that BrdU could induce many biological responses of genotoxicity, such as locus mutation and the fragile sites expression in the human genome [16]. It is reported that long-term BrdU treatment for 6 d significantly increased the level of the alpha V-associated integrin and inhibited cell growth in both culture medium and soft agar [17]. However, short-term exposure to BrdU is relative safe and may have less harmful biological reactions. For example, there was no evidence of the anti-mitogenic action of BrdU after 18 h incubation with CD^4+^ T cells [18], which suggested that short-term treatment with BrdU did not disturb the cell cycle. The study showed that BrdU (10, 60 and 120 mg/ml for 4 d) had no cytotoxic effects on hippocampal cells and immature neurons proliferation [19]. Moreover, low concentration of BrdU had no inhibitory effect on adult neural progenitor cells [20] and did not prevent the differentiation of preadipocyte [21]. In the present study, the level of BrdU used was 10 μM and the incubation time was only 2 h before the WST-1 measurement, which may account for the lack of disturbance found on cell viability.

The chemical nature of WST-1 is the second component allowing the possible simultaneous measurements of these two reagents. WST-1 is a light-red, highly sensitive tetrazolium that produces water highly soluble formazan after the NADPH oxidase reduction in mitochondria. Because WST-1 is different from the cell-permeable MTT, which produces insoluble formazan that accumulates inside cells, the toxicity of WST-1 to cells is significantly decreased. It is very likely that the non-toxic WST-1 is compatible with BrdU and the same cells can be used to conduct the subsequent experiments. Our study showed that the result of the BrdU assay performed after the WST-1 measurement was consistent with the result obtained from the direct BrdU examination, which confirmed that WST-1 did not affect cell proliferation.

## Methods

### Chemicals and reagents

All of the chemicals used were of analytical grade. The BrdU cell proliferation ELISA kit was purchased from Roche Applied Science, the WST-1 cell viability kit was purchased from Beyotime Biotechnology Inc. The other important components were the following: DMEM (DMEM Dulbecco's Modified Eagle Medium, Hyclone), FBS (fetal bovine serum, Gibco), 1 × PBS (phosphate buffered saline, Hyclone), 0.25% trypsin (Sigma), 10,000 U/ml penicillin and 10,000 μg/ml streptomycin (Hyclone), 100 cm^2^ cell culture dishes (Corning), 96-well plate (Corning), spectrophotometer (Bio-tek). The purified MRP-14 (migration inhibitory factor-related protein 14) recombinant protein was used as the testing reagent in the study [22].

### Cell preparation and culture

The isolation and culture of the airway smooth muscle cells (ASM cells) were performed according to the previous description [23]. Briefly, after redundant tissue dissection and washing of the rat trachea in sterile, ice-cold, HPPS solution (10.0 mM HEPES, 130.0 mM NaCl, 5.0 mM KCl, 1.2 mM MgCl_2_ · 6H_2_O, 10.0 mM glucose, pH = 7.4), enzyme digestions (2.0 mg/ml collagenase IV and 0.05% elastase) was conducted for 30 min at 37°C. The pellet was then resuspended and cultured in DMEM with 10% FBS. The ASM cells were confirmed by the immunofluorescence of SM α-actin. The culture medium was renewed every 2–3 days and experiments were performed with cells at passages 3–10.

### The study design

**Figure 2:**
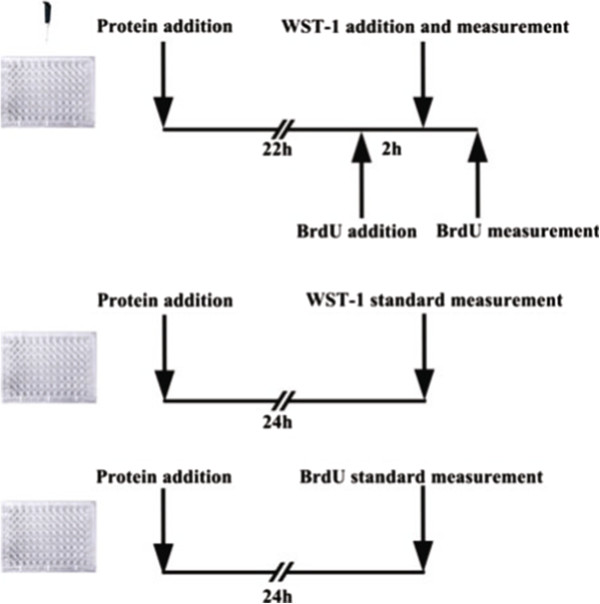
**Schematic of the simultaneous BrdU and WST-1 measurements.** The MRP-14 protein was added at concentrations of 1, 10, 50, 100, 200, 400 and 800 ng/ml. Before adding the WST-1 reagent into the plate at the time point of 24 h, the BrdU was added and incubated for 2 h. After the WST-1 measurement was conducted, the BrdU assay was performed.

The MRP-14 protein was given at doses of 1, 10, 50, 100, 200, 400, 800 ng/ml (final concentration, the same as below) into the 96-well plate and incubated for 24 h. BrdU (10 μl/well, 10 μM) was added into the plate 2 h before the WST-1 measurement in order to maintain BrdU in the well for 4 h. 10 μl WST-1 was added at the time point of 24 h, then incubated for 2 h and measured. After the WST-1 measurement, the BrdU examination was performed according to the manufacturer’s instructions. The schematic of the simultaneous BrdU and WST-1 measurements was showed in Figure 2.

### BrdU measurement

The BrdU measurement was conducted according to the standard protocol of manufacturer. After culturing the ASM cells (5000/well) in the 96-well plate overnight, the MRP-14 protein was given to obtain final concentrations of 1, 10, 50, 100, 200, 400, 800 ng/ml and incubated for 24 h. BrdU (10 μl/well, 10 μM) was added and incubated for 4 h at 37°C. The medium was moved by tapping. The cells were then fixed and incubated for 30 min at room temperature. After thorough removal of the fix solution by tapping, 100 μl/well working solution of BrdU antibody conjugated with peroxidase was added and incubated for 90 min at room temperature. The wells were rinsed three times with the washing solution (200 μl/well), and the substrate solution (100 μl/well) was added. The absorbance was monitored at 370 nm.

### WST-1 measurement

The WST-1 measurement was performed according to the standard protocol of manufacturer. Briefly, the ASM cells (5000/well) were cultured into the 96-well plate and incubated overnight. Then the MRP-14 protein was added to obtain final concentrations of 1, 10, 50, 100, 200, 400, 800 ng/ml and incubated for 24 h. 10 μl WST-1 was added and incubated for another 2 h at 37°C in the incubator. The absorbance was monitored at 450 nm.

### Statistics

The Statistical significances among the different groups of the BrdU and WST-1 measurements were calculated using one-way ANOVA followed by LSD posthoc test. P value less than 0.05 was considered significant.

## Conclusion

The study demonstrated that BrdU and WST-1 measurements can be applied simultaneously without mutual interference, which may be useful for the cellular functions examination and efficacy improvement.
